# From Chitosan to Chitin: Bio‐Inspired Thin Films for Passive Daytime Radiative Cooling

**DOI:** 10.1002/advs.202206616

**Published:** 2023-02-15

**Authors:** Tobias Lauster, Anika Mauel, Kai Herrmann, Viktoria Veitengruber, Qimeng Song, Jürgen Senker, Markus Retsch

**Affiliations:** ^1^ Department of Chemistry Physical Chemistry I University of Bayreuth Universitätsstraße 30 95447 Bayreuth Germany; ^2^ Department of Chemistry Inorganic Chemistry III and Northern Bavarian NMR Center University of Bayreuth 95447 Universitätsstraße 30 Bayreuth Germany; ^3^ Department of Chemistry Physical Chemistry I Bavarian Polymer Institute Bayreuth Center for Colloids and Interfaces and Bavarian Center for Battery Technology (BayBatt) University of Bayreuth Universitätsstraße 30 95447 Bayreuth Germany

**Keywords:** absorption spectroscopy, broadband optical characterization, passive cooling, solid‐state nuclear magnetic resonance spectroscopy, thermal emission

## Abstract

Passive radiative daytime cooling is an emerging technology contributing to carbon‐neutral heat management. Optically engineered materials with distinct absorption and emission properties in the solar and mid‐infrared range are at the heart of this technology. Owing to their low emissive power of about 100 W m^−2^ during daytime, substantial areas need to be covered with passive cooling materials or coatings to achieve a sizeable effect on global warming. Consequently, biocompatible materials are urgently needed to develop suitable coatings with no adverse environmental impact. It is shown how chitosan films with different thicknesses can be produced from slightly acidic aqueous solutions. The conversion to their insoluble form chitin in the solid state is demonstrated and the conversion is monitored with infrared (IR) and NMR spectroscopy. In combination with a reflective backing material, the films show below‐ambient temperature cooling capabilities with a suitable emissivity in the mid‐IR region and low solar absorption of 3.1–6.9%, depending on the film thickness. This work highlights the potential of chitosan and chitin as widely available biocompatible polymers for passive radiative cooling applications.

## Graphical abstract

The potential of chitosan and chitin for the application as passive radiative cooling materials is investigated. A solid‐state reaction is used to convert chitosan to its insoluble form chitin. Based on extensive optical characterization, both biopolymers are capable of below ambient temperature passive cooling.

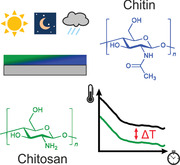

## 1 Introduction

With the prospects of global warming, the demand for cooling applications is expected to increase in the future. In classical active cooling technologies like air conditioning, the heat is spatially displaced but remains in close vicinity. Furthermore, the AC unit releases excess waste heat into the environment. This leads to heat accumulation, especially in urban areas. Therefore, developing new materials to save energy for cooling applications is of great interest. One promising concept to mitigate the heat island effect can be found in materials that cool in a passive manner. By tuning the optical properties of a material such that it efficiently emits energy in the form of thermal radiation, cooling can be achieved without energy input during operation.

This potential for cooling applications was already identified and theoretically described several decades ago.^[^ ^1^ ^]^ Passive radiative cooling materials dissipate heat into outer space. For this heat sink to be accessible, the radiation needs to pass the atmosphere, which is only partially transparent. The most crucial wavelength region is located in the mid‐infrared (mid‐IR) region between 8 and 13 µm, where the atmosphere is mostly transparent, and blackbody radiation at typical ambient temperatures (298 K) shows an emission maximum. If a material emits energy in only partially transparent or opaque wavelength regions, it depends on the temperatures of the material and the atmosphere whether cooling occurs.

During the daytime, sunlight absorption must be prevented to provide a net cooling effect. Beginning with the first works that demonstrated cooling under direct sunlight illumination,^[^ ^2^ ^]^ the field of passive radiative daytime cooling significantly developed over the last decade.^[^ ^3^ ^]^

Polymeric materials such as polydimethylsiloxane^[^ ^4^ ^]^ or fluorinated polymers^[^ ^5^ ^]^ are heavily investigated because they show beneficial absorption (and therefore emission) characteristics in the mid‐IR region due to resonance of molecular vibrations. At the same time, those materials absorb very little sunlight. In combination with a metallic reflection layer to prevent sunlight absorption of the underlying substrate, simple and low‐cost passive radiative coolers were realized. Other works combine inorganic particles with a polymeric matrix to achieve the desired optical properties^[^ ^6^ ^]^ or utilize metal‐dielectric multilayer photonic structures to tune the absorption and reflection.^[^ ^7^ ^]^ The use of metallic reflection layers can even be omitted if the material sufficiently backscatters the sunlight. This was shown in recent works, where porosity was introduced to the systems to enable scattering.^[^ ^5a^ ^]^ Besides synthetic materials, bio‐based materials have been researched. Especially, cellulose‐based materials like wood or cellulose fibers drew increasing attention because they are abundant, low cost, and have a minimum environmental impact.^[^ ^8^ ^]^ Cellulose derivatives like cellulose acetate showed remarkable scattering properties when structured, as demonstrated in recent works.^[^ ^9^ ^]^ Also, natural silk showed cooling capabilities with strong reflectance attributed to Anderson localization within the fibers.^[^ ^10^ ^]^ Another natural source of inspiration has been the Silver Sahara Ant for passive daytime cooling concepts and materials.^[^ ^11^ ^]^ An intricate multiscale structure of nano‐corrugated prismatic fibers based on chitin (CT) was shown to cool the Silver Sahara Ant by reflection in the solar range and enhanced emission in the mid‐IR range. Furthermore, chitosan (CS) or its acetylated form CT is a material that can form structures with extraordinary optical properties. For example, the scales of the *Cyphochilus* beetle exhibit whiteness due to light scattering at the random CT network structure.^[^ ^12^ ^]^ A property that is also very desirable for passive radiative cooling materials. CS is, after cellulose, the second most abundant polymer in nature but has received very little attention for passive radiative cooling. Due to the polycationic nature of the polymer, it has been shown that CS sponges are excellent for the adsorption of anionic pollutants.^[^ ^13^ ^]^ Further applications are found in medical fields as support for tissue engineering,^[^ ^14^ ^]^ as a sensor for bacteria detection,^[^ ^15^ ^]^ or as template source for the fabrication of synthetic nacre.^[^ ^16^ ^]^ Besides the abundance, CS has the advantage of being soluble in dilute acetic acid, enabling the processing from solution. However, CS's potential for passive radiative cooling remains widely unexplored. An exception is the work of Chang et al., who used electrophoretically deposited CS to alternate the emission properties of stainless steel.^[^ ^17^ ^]^ Also, the group around Liu et al. used CS as a matrix material for lignin nanoparticles to produce a photothermal film but focused on the UV–vis absorption properties within their study.^[^ ^18^ ^]^

Our work demonstrates the capabilities of CS as passive cooling material. To make it useable for passive cooling applications under outdoor conditions, CS must be transformed into CT. CT is the sturdy and water‐insoluble form prevalent in nature, whereas CS is necessary to allow for processing it from solution. We, therefore, focus in this contribution on the processing and transformation of CS into CT with a particular focus on its broadband optical properties and passive cooling performance.

## 2 Results and Discussion

The CS films investigated in this work were cast from solutions of CS powder in dilute acetic acid. After casting, an aqueous gel film forms on the substrate, and compact CS films remain after evaporation of the solvent. With this approach, a variety of film thicknesses can be realized. The cast solution can be spread by doctor blading to obtain thin films. Several layers of CS can be stacked for thicker films by adding another layer of solution after drying the previous layer. The overall thickness can be controlled by the amount of solution and the concentration. However, the concentration regime that can be used is limited to low concentrations (<4 wt%) because the viscosity of the solution is strongly increasing for higher concentrations. With the mentioned techniques, film thicknesses ranging from 6 to >100 µm can be prepared. With the spin coating technique, even thinner films can also be realized.

### 2.1 Acetylation Reaction to CT

The advantage of water processability of CS becomes a drawback when considering outside applications as passive cooling material. Water or humidity exposure will lead to the destruction of the material and its functionality. We performed a re‐acetylation reaction to counter this disadvantage and to render the material insoluble. We followed the procedure of Hirano et al.,^[^ ^19^ ^]^ in which the reaction of a CS fiber with acetic anhydride (AcAH) in methanol is performed. The proposed reaction can be done at room temperature, and the material's structure is retained. A detailed description of our reaction conditions can be found in the Experimental Section.

#### 2.1.1 Degree of Acetylation

**Figure 1:**
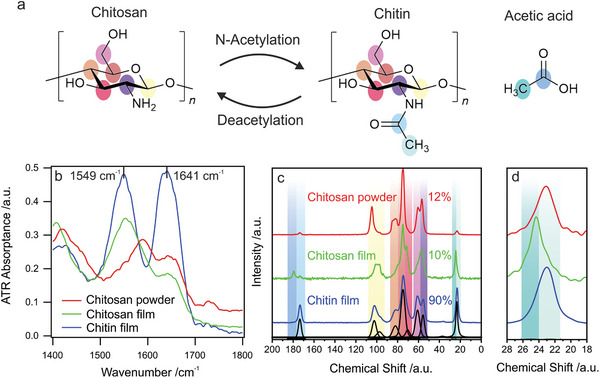
a) Chemical structure of chitosan (Poly‐d‐Glucosamine), chitin (*N*‐Acetyl Poly‐d‐Glucosamine), and acetic acid. b) Normalized ATR‐FTIR spectra were collected from CS powder, a CS film, and after the conversion to CT. The vibrational resonances corresponding to —NH at 1549 cm^−1^ and the amidic carbonyl resonance at 1641 cm^−1^ are marked. A slight difference in the intensity ratio between the NH deformation and amidic carbonyl stretching vibration for the chitosan powder and film indicates a different alignment and stronger involvement in hydrogen bonding for the chitosan films. This is in agreement with the observed shift in the resonance frequency of the NH‐deformation vibration. The relative increase of the amide absorptions indicates a conversion from CS to CT. c) ^1^H^13^C CP MAS NMR spectra of CS powder, CS films, and a film after the conversion reaction with a color‐coded signal assignment. The spectra are normalized to the backbone resonances (C1‐C6: 120–40 ppm) and shifted vertically for a better overview. Upon treatment with AcAH, the DA increases. Exemplarily, the refinement of the CT film spectrum is shown (black). Intensities derived from the refinements are given in Table S1, Supporting Information. d) Enlarged section of the ^13^C CP MAS NMR spectra to show the different CH_3_ resonances of CT and acetic acid. The spectra are normalized to the CH_3_ resonances (28–18 ppm) and shifted horizontally for a better overview.

To characterize if the reaction was successful, we determined the degree of acetylation (DA) via IR and NMR spectroscopic measurements. The DA describes the relative amount of *N*‐acetyl glucosamine units within the polymer. CS mainly consists of glucosamine units. Consequently, DA values are low. In contrast, the polymer is named CT for DA values above 50%. The chemical structures of the predominant repeating units in each of the two polymers are displayed in **Figure** **1**a.

NMR and FTIR spectroscopy are complementary, non‐destructive methods to determine the DA. NMR spectroscopy is an element‐sensitive bulk method that enables quantitative characterization. In contrast, FTIR spectroscopy is fast and requires minimal sample preparation, but has limited sample penetration depth when the attenuated total reflection (ATR) technique is used. Several procedures have been proposed to determine the DA from IR spectra. However, the evaluation can lead to deviations depending on the chosen probe and reference signals. An overview can be found in the work of Duarte et al.,^[^ ^20^ ^]^ in which they compare the results of different evaluation procedures of IR spectra to the DA values obtained with NMR spectroscopy. Nevertheless, ATR‐FTIR spectroscopy is well suited to gain qualitative insights into CT conversion.

Particularly the —NH vibrational mode and the amidic carbonyl stretching vibration serves as an indicator of acetylation. According to the literature, the corresponding absorptions are found at wavenumbers of 1561 and 1663 cm^−1^, respectively.^[^ ^20^ ^]^ Before functionalization, the‐ NH absorption was more pronounced than the amidic carbonyl absorption in both the pristine CS powder and film sample (Figure 1b). In contrast, similar intensities are observed after the functionalization reaction with AcAH. The absorption increase from the amidic carbonyl groups indicates that CS was acetylated. Note that the actual peak positions (1549 and 1641 cm^−1^) slightly deviate from literature values. This deviation can result from different orientations or microstructure of our actual polymer. The collected spectra for the full wavenumber range are shown in Figure S1, Supporting Information. Theoretically, the hydroxyl groups within the polymer could also react with AcAH. However, the carbonyl vibration of the resulting esters would be expected at wavenumbers >1700 cm^−1^, where no new signals arise.

To confirm our qualitative observations from FTIR spectroscopy and extend these to a quantitative level, we performed solid‐state NMR spectroscopy. While IR spectra of the film samples prepared on glass substrates could be measured directly, the films needed to be detached from the substrate, cryomilled, and packed into magic‐angle spinning (MAS) rotors prior to NMR spectroscopic measurements. The resulting ^13^C CP MAS NMR spectra of pristine CS powder and a CS film before and after the acetylation reaction are displayed in Figure 1c,d. The signals corresponding to the glucosamine backbone (C1‐C6) of the polymer are located between 110 and 50 ppm. Chemical shifts and signal shapes vary with local molecular conformations and, consequently with the degree of crystallinity and the present polymorphs. Generally, the crystallinity increases with the DA.^[^ ^21^ ^]^ Additional to the backbone signals, peaks at 174 ppm and 23 ppm are present in all spectra. These signals correspond to the carbonyl carbon and methyl carbon in the *N*‐acetyl group, respectively. A significant increase in their intensity upon acetylation is observed, confirming the qualitative IR spectroscopic results. Small amounts of residual acetic acid (180 ppm for the carboxyl function and 25 ppm for the methyl group) were observed in the CS film sample prior to functionalization. The films are prepared by dissolving the pristine CS powder in dilute acetic acid, followed by drying on glass substrates and removal of residual acetic acid via treatment with NaOH in methanol (see Experimental Section). The ^13^C CP MAS NMR spectra show that the removal of acetic acid was incomplete. However, those additional signals vanish after the acetylation reaction, indicating that no acetic acid remains within the final film.

To quantify the DA of our product, CP NMR spectra were measured with a set of optimized parameters as previously determined in detailed relaxation and CP kinetics studies by Duarte et al.^[^ ^22^ ^]^ (see Experimental Section). They ensure similar intensities for the resonances of primary, secondary, and tertiary carbon units, thus avoiding the drawback of a CP pulse sequence not necessarily leading to quantitative results.^[^ ^23^ ^]^ Still, the resonance intensities of carbon units without covalently attached protons will be underestimated as the magnetization transfer from ^1^H to ^13^C in a CP sequence is achieved via heteronuclear dipolar interactions and consequently depends on the proximity of protons.^[^ ^24^ ^]^ Thus, the DA needs to be determined as the ratio of the methyl signal of acetic acid to the six backbone carbon atoms. After deconvolution of the spectra with pseudo‐Voigt profiles (Figure 1d and Table S1, Supporting Information), we calculated DA values. It is important to consider the residual acetic acid to prevent overestimation of the DA. For the pristine CS powder and the unreacted film, we determined low DA values of 12% and 10%. After the reaction, a DA value of 90% confirms successful acetylation and conversion of the film to CT.

#### 2.1.2 Water Resistance and Contact Angle

**Figure 2:**
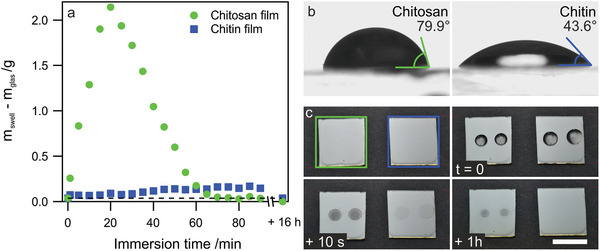
a) Mass change of CS and CT films after consecutive water immersion for 90 min and after complete drying. b) The water contact angle of CS and CT film was determined in a sessile drop experiment. The presented values are mean ± SD. c) Photographs of CS and CT films before and at different times after water droplet exposure. The scale bar is 2 cm.

The main goal of the acetylation reaction was to increase the water resistance of the films. We prepared a set of CS and CT samples on glass substrates to compare the dissolution behavior. The weight of the sample *m*_swell_ (sample + substrate) was monitored after a certain immersion time in the water for each sample, and the mass difference to the substrate (*m*_glass_) was calculated. The results of this test are shown in **Figure** **2**a. The mass difference is strongly increasing for the CS film within the first 20 min of immersion. During the experiment, a strong swelling of the film can be observed, and we attribute the increase in mass to this swelling. Also, a mass increase can be observed for the CT film, but this increase is much less pronounced. For longer immersion times, the mass of the CS film starts to decrease, indicating dissolution or detachment of the film. After 90 min of the experiment, only a minor fraction is left. The last data points in Figure 2a were recorded after drying the samples overnight. The experiment reveals that the CT film is completely recovered, and the initial mass is restored (dashed line), while the CS film was completely removed during the experiment.

The significant differences between CS and CT are further highlighted by water contact angle measurements in a sessile drop experiment (Figure 2b). The contact angle of water strongly decreases upon transformation to CT and presents a more hydrophilic surface despite its increased water resilience. We noticed after the experiment that the sample surface of the pristine CS film could not be recovered due to the partial dissolution of the film. Figure 2c shows a time series of photographs showing the samples before and after water droplet exposure. In contrast, the acetylated film can fully recover to its original state.

### 2.2 Optical Characterization

**Figure 3:**
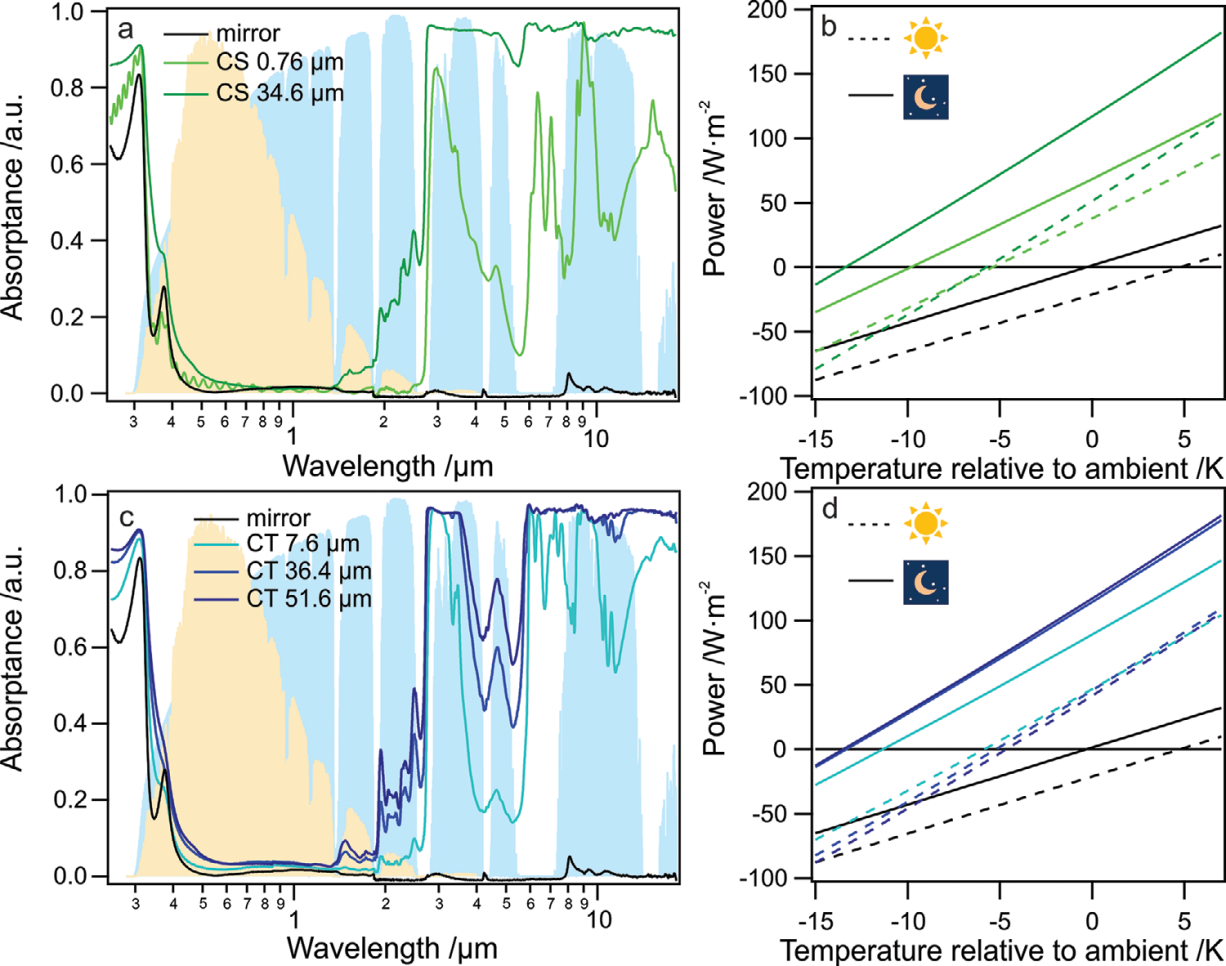
Absorptance of a) chitosan films (CS) and c) chitin films (CT) with different thicknesses on silver mirror substrates. As a reference, the absorptance of the plain silver mirror is shown. For comparison, the atmospheric transmittance (blue) and an AM 1.5 solar spectrum (yellow) are shown in the background. Calculated cooling power as a function of temperature below ambient for b) CS films and d) CT films with different thicknesses for daytime (dashed lines) and nighttime (solid lines). The intersection at zero cooling power represents the equilibrium temperature below ambient that can be reached.

To test the cooling potential of our films, we chose a reflective silver mirror as an underlying substrate to ensure high solar reflectivity. The material's absorption (and consequently emission) properties strongly depend on the thickness of the film. For comparison, we prepared samples with different thicknesses and measured the broadband optical properties between 0.25 and 18.2 µm (**Figure** **3**a). This wavelength range covers the entire solar and the mid‐IR range. The absorptance was calculated from integrated reflectance measurements considering energy conservation with 1 − reflectance. Transmittance can be neglected due to the underlying silver mirror substrate. As a reference, we plotted the absorptance of the plain mirror substrate (black line).

For comparison, an AM 1.5 solar emission spectra and the atmospheric transmittance regions are displayed in the background. Increasing film thickness from 0.76 to 34.6 µm has only minor effects within the solar spectral region. The strong absorption toward UV wavelengths is a combination of absorption of the underlying Ag substrate and CS itself since the absorptance increases with material thickness, but it is also present for the plain Ag substrate. Toward the NIR regime, more pronounced solar absorption occurs for the thicker sample. We calculated the overall solar absorption based on the collected spectra and the displayed AM 1.5 solar spectrum (details in experimental). We found solar absorption of 3.1% and 6.6% for the thin and thick samples, respectively. Based on the low solar absorption, both samples are capable of exhibiting below ambient radiative daytime cooling.

In the IR region, distinct peaks are visible for the thin sample, which are only partially located within regions of atmospheric transparency. With increasing thickness, the absorptance is more comparable to a broadband emitter. We expect a cooling power characteristic between the selective and broadband cases for thin samples.^[^ ^3a^ ^]^ Besides the absorptance in the UV region, the silver mirror substrate also shows minor absorptance in the mid‐IR region. Those can be attributed to the presence of a glassy protection layer on the mirror surface.

To further elucidate the cooling characteristics of the two samples, we calculated the theoretical temperature reduction based on the measured absorptance (Figure 3b). Details about the calculations can be found in the Experimental Section. In short, we considered an AM 1.5 solar spectrum with irradiation of 1000 W m^−2^, the atmospheric transmission spectrum modeled with Modtran, an ambient temperature of 25 °C, and a non‐radiative heat transfer coefficient of *h* = 4.4. Wm^−2^ K^−1^. For the daytime case (dashed lines), we found that both samples should reach almost comparable temperatures. In contrast, for the nighttime case (solid lines), the thicker sample should reach lower equilibrium temperatures (temperature at *P* = 0 Wm^−2^). Our calculations further reveal that the silver substrate is expected to heat above ambient temperature during the daytime. However, in combination with the CS films, both samples reach below ambient temperatures in the calculations.

We also prepared a set of CT films and measured the absorptance. Unfortunately, the direct preparation and reaction on the mirror substrates were unsuccessful since the films deformed and detached during the reaction. We, therefore, prepared the films on separate glass plate substrates, performed the reaction, and attached the CT films afterward to the mirror substrates. Note that we did not succeed in the preparation of very thin (<5 µm) CT films of sufficient macroscopic size because they ruptured during the transfer process. The absorptance spectra of the CT films (Figure 3c) are comparable to the previously discussed CS spectra. Especially the two films with ≈35 µm thickness show a similar low solar absorptance with 6.6% for CS and 6.9% for CT, respectively. A direct comparison of spectra collected from the films without the silver substrate is shown in Figure S2, Supporting Information.

We also calculated the expected cooling performance for CT films (Figure 3d) and found that during nighttime operation, thicker CT films reach similar below ambient temperatures as found for CS. All CT samples are expected to reach similar below ambient temperatures during the daytime.

### 2.3 Passive Cooling Properties

**Figure 4:**
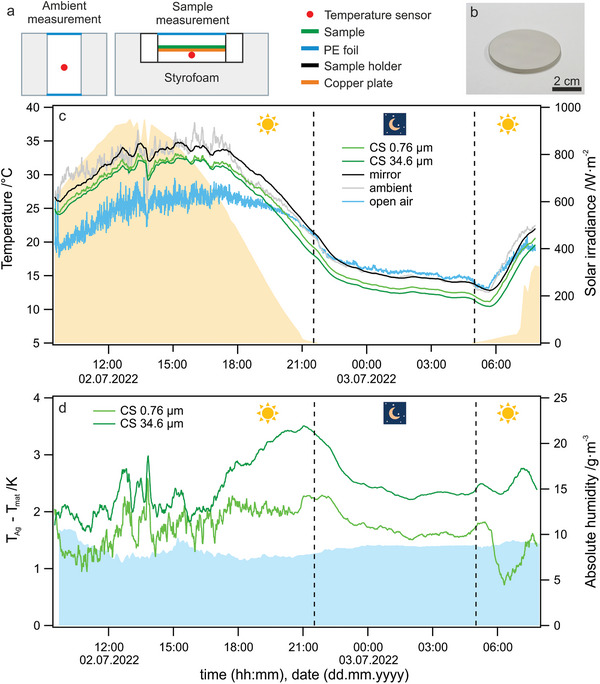
a) Scheme of the measurement environment of the ambient reference and the passive cooling sample temperature. The sample (dark green line) is a layered construction with CS being directly deposited on a quartz glass substrate coated with a silver layer shown in b). The sample is placed on a copper plate (orange line) to ensure an even temperature distribution. The temperature measurement of the sample is conducted at the underside of the copper plate. c) Temperature measurement for CS samples of different thicknesses on mirror substrates over a day and night cycle. A plain mirror substrate, the ambient temperature, and the open air temperature are shown as references. The solar irradiance during the measurement is displayed in the background. d) Temperature difference of the CS samples compared to the plain silver mirror over the entire measurement period. In the background, the absolute humidity (blue) during the measurement is shown, confirming constant atmospheric conditions.

To confirm the calculated cooling performance, we conducted a rooftop experiment with the CS samples on mirror substrates and measured the temperature over a full day and night cycle (from 09:30, 9th to 08:00, 10th of July 2022, University of Bayreuth, Bayreuth, Germany). The setup is shown in **Figure** **4**a and Figure S3, Supporting Information. We used polystyrene foam insulation to reduce conductive losses to the surrounding air and an LDPE cover foil to reduce convective losses, respectively. The entire setup was covered with Al foil to reflect solar radiation. The sample (Ag mirror with coated CS layer, Figure 4b) was placed on a thin copper plate inside the Styrofoam chamber, ensuring a good temperature distribution, which was monitored with a thermocouple directly at the underside of the copper plate.

The resulting temperature measurements (Figure 4c) confirm a below‐ambient temperature for both samples for the nighttime and daytime cases. The ambient temperature is measured in a reference box without convective losses and without any other optical components other than the styrofoam container and the LDPE cover. Comparable to the silver mirror reference, we observe a temperature increase during the daytime of about 8 °C relative to the open‐air temperature. We attribute this to parasitic solar absorption by the sample holder and a resulting greenhouse effect within the measurement and reference chamber.^[^ ^25^ ^]^ The Ag mirror absorption in the UV range (Figure 3a,c) apparently has a minor effect on the temperature in the sample chamber since the ambient and Ag mirror reference temperature coincide. Considering the CS coated samples, we observe that the thicker (more broadband emitter) sample reaches lower overall temperatures, indicating that the additional cooling power gained with increasing thickness is overweighing the solar absorption (as discussed in Section 2.3). This trend is in line with the expectation based on the calculation in Figure 3b. We attribute the quantitative difference between the calculation and experiment to the actual setup's unknown non‐radiative heat loss coefficient and the additional heat input caused by the aforementioned greenhouse effect within the measurement box. Additionally, we calculated the experimental temperature difference between the samples and the silver mirror reference (Figure 4d) to highlight the contribution of the CS layers. Both samples are colder than the mirror, and the thicker sample reaches up to ≈3.5 °C lower temperatures. We calculated the absolute humidity from relative humidity measurements, which remained almost constant for the entire measurement duration. The higher variability of the temperature reduction relative to the silver mirror reference during daytime is, consequently, caused by subtle changes to the solar radiance influx on the cooling films.

Based on the optical similarity between CS and CT film (see Figure 3a,c) one can expect a comparable cooling performance in the case of CT films. However, the direct measurement of the temperature reduction is affected by the increased brittleness and detachment of the CT film, which renders accurate temperature measurements difficult in this case.

## 3 Conclusion

In conclusion, we outlined the potential of CS and CT as biocompatible polymers for passive cooling applications. Therefore, we presented how CS films of different thicknesses can be produced from a slightly acidic aqueous solution. We demonstrated that the films can be converted to CT within the solid state. We confirmed the selective *N*‐Acetylation of CS with solid‐state ^13^C CP MAS NMR spectroscopy and FTIR‐spectroscopy and determined the degree of acetylation. Our presented approach can evaluate the DA even when residual free acetic acid remains in the sample. We further show that the conversion to CT strongly enhances the film's water resistance, a property especially important for outdoor applications as passive cooling material. To evaluate the passive cooling potential, we used silver mirror substrates as reflectors and measured the absorption properties of the films with UV–vis and FTIR‐spectroscopy. CS and CT have very similar optical properties and excel with low solar absorptance in combination with good IR‐emission. Based on the optical properties, we calculated the potential cooling power and found that both materials can show below ambient radiative daytime cooling without adding fillers. We demonstrate the cooling potential of CS in a field test and measure reduced temperatures compared to the plain silver mirror for day and nighttime. We believe that these natural polymers expand the toolbox of available materials to realize passive cooling systems. Especially factors like abundance, water processability, biocompatibility, and biodegradability are clear advantages, and we encourage other researchers to utilize those.

## 4 Experimental Section

### Materials

CS (medium molecular weight, Aldrich), acetic anhydride (>99% for synthesis, Carl Roth chemicals), glacial acetic acid (Merck), and sodium hydroxide (Sigma Aldrich) were used without further purification. Methanol (VWR chemicals) was dried over a molecular sieve with a pore diameter of 3 Å before use. Protected round silver mirrors (2" Ø, Thorlabs)

### Preparation of Films

Films with different thicknesses were produced by drying a certain amount of solution on a glass or silver mirror substrate in air. For the formation of thick films, 3–6 g of a 3 wt% solution of CS in diluted acetic acid (1 v%) were poured on the substrate and left to dry. For thin films, the solution was spread by doctor blading with a slit size of 400 µm. To access intermediate thicknesses, the doctor blading process was repeated upon drying of a previous layer. A 0.79 µm CS film was produced by spin coating of a 3 wt% solution on the silver mirror substrate at 3000 rpm for 90 s and left to dry in air.

### Acetylation to CT

An acetylation reaction was performed with a procedure described by Hirano et al.^[^ ^19^ ^]^ to increase the degree of *N*‐acetylation. Before the reaction, the CS films were washed with 0.5 m NaOH dissolved in methanol to remove acetic acid, followed by pure methanol to remove the excess base. The film was immersed in methanol, and acetic acid anhydride was added (5 mol/mol Glucosamine) and stirred overnight. The films were washed with 0.5 m NaOH solution in methanol and pure methanol and then dried with compressed air. The resulting films were stored in a desiccator over saturated NaCl solution in water for at least 1 h, with the aim to reduce mechanical stress within the film. The so‐produced film was transferred to a silver mirror substrate (protected round silver mirror, Thorlabs) for the cooling experiments.

### Optical Characterization

UV–vis spectroscopy was performed with a Cary 5000 spectrometer (Agilent Technologies, Germany) for the wavelength range from 250–2500 nm. The total reflectance was measured with an integrating sphere accessory (Labspheres) at the respective port of the sphere. As a reference, a Spectralon white standard (Labspheres) was used. The absorptance of the samples was calculated considering energy conservation with 1 − reflectance. Transmittance can be neglected because the silver mirror prevents any light transmission. The relative solar absorptance was calculated by multiplication of the measured absorptance *A* (*λ*) with the solar radiance of the AM 1.5 spectrum *I*_AM1.5_ (*λ*) and divided by the total radiance $\frac{A\left(\lambda \right)*I_{\mathrm{AM}1.5}\left(\lambda \right)}{I_{\mathrm{AM}1.5}\left(\lambda \right)}$.

FTIR spectroscopy was performed with a Vertex 70 spectrometer (Bruker) combined with a gold‐coated integrating sphere accessory (A562, Bruker). The reflectance was measured in the wavelength range from 1.33–18.3 µm at the respective port of the sphere. As a reference, a gold mirror was used. The absorptance was calculated with 1 − reflectance, neglecting transmission due to the silver mirror below the samples.

### Degree of Acetylation

The acetylation reaction was confirmed by ATR‐FTIR spectroscopy using a Platinum ATR diamond accessory (A225/Q, Bruker) in combination with a Vertex 70 IR spectrometer (Bruker).

### Solid State NMR Spectroscopy

The degree of acetylation of the CS samples was monitored via ^13^C CP MAS NMR spectroscopy. The film samples were ground with the CryoMill SPEX CertiPrep 6750 (C3 Prozess‐ und Analysentechnik) and packed into 3.2 mm zirconium rotors. The spectra were recorded using a Bruker Avance‐III HD spectrometer operating at a B_0_ field of 9.4 T (*ν*_0_(^1^H) = 400.01 MHz and *ν*_0_(^13^C) = 100.62 MHz), a 3.2 mm triple resonance probe (Bruker) and a spinning speed of 13.75 kHz. The used pulse sequence consisted of presaturation pulses, a proton 90° pulse (*ν*_nut_(^1^H) = 100 kHz), and a ramped ^1^H‐^13^C CP sequence (*ν*_nut_(^13^C) = 41 kHz and 47 kHz ≤ (*ν*_nut_(^1^H) ≤ 67 kHz) with a contact time of 1.0 ms. This contact time was chosen for semi‐quantitative measurements according to Duarte et al.^[^ ^22^ ^]^ During acquisition, proton broadband decoupling with a spinal‐64 sequence (*ν*_nut_(^1^H) = 70 kHz) was applied. The spectra were referenced to tetramethylsilane (TMS) using the secondary standard adamantane. The signal assignment was performed according to the literature^[^ ^22^, ^26^ ^]^ and was aided by ^13^C NMR prediction packages included in ChemSketch (ACD/Labs). To deconvolute the spectra with pseudo‐Voigt profiles, the simulation package SOLA, included in TopSpin 3.2 (Bruker), was used. After deconvolution (Table S1, Supporting Information), the DA was given by the ratio of methyl carbons (CH_3_) to CS repeating units. One CS repeating unit consisted of 6 carbons, thus the DA was calculated as follows.

(1) $$\mathrm{DA}=\frac{n\left({\mathrm{CH}}_{3}\right)\;\times \;6}{n\left(\mathrm{backbone}\right)}$$

### Water Contact Angle

The water contact angle was measured with a Contact Angle System OCA (DataPhysics Instruments GmbH) in sitting drop geometry. A 10 µL droplet was placed on the solid/air interface, and a picture was collected from side view with a camera. The contact angle was determined graphically at the three interface (solid/liquid/gas) contact points.

### Calculation of Passive Cooling Performance

The calculated cooling power was based on the measured optical properties. Following the approach presented previously,^[^ ^27^ ^]^ an AM1.5 solar spectrum and an atmospheric transmittance spectrum generated for the measurement location (Bayreuth) using Modtran were employed. The non‐radiative heat transfer coefficient was assumed to be 4.4 W m^−2^ K^−1^, while the atmospheric temperature was taken as 298.15 K. The angle‐dependent emissivity is, furthermore, considered to be Lambertian diffuse. All integrations were done using the trapezoidal integration method in Matlab. As functions inside the integrals need to have the same energy spacing to perform numerical integration, the sun's and atmosphere's spectral radiative powers were adapted to the spacing of the measured emissivity because they were available in higher resolution. This adaptation was made using the 1D data interpolation method in Matlab.

### Outdoor Passive Cooling

The rooftop experiments were conducted in a self‐built measurement setup on the roof of a four‐floor building at the university of Bayreuth (Germany) under a clear sky. The samples and mirror reference were placed in a 3D printed sample holder (acrylonitrile butadiene styrene) on top of a copper plate for temperature distribution. A Pt100 thermocouple below the copper plate was used for temperature recording with a digital multimeter (DAQ6510, Tektronix, Germany) every 5 s. The sample holder was placed in a Styrofoam box for thermal insulation to the surroundings, and an LDPE foil was used to cover the sample and reduce convection. The entire Styrofoam box was covered with mylar aluminum foil to ensure high solar reflectance. The ambient temperature was recorded by a Pt100 thermocouple enclosed in a similar Styrofoam box with LDPE foil covered on top and bottom to allow sunlight transmission but include the parasitic heating within the box in the ambient reference measurement. The weather data were obtained from the weather station at the University of Bayreuth (Ecological‐Botanical Garden, 400 m away from the experiment).

The relative humidity RH was tracked next to the setup with a temperature logger (LOG220, DOSTMANN electronic GmbH). The absolute humidity AH was calculated with RH in % and the ambient temperature *T*_amb_ in °C with $\mathrm{AH}=\frac{6.112\cdot e^{\frac{17.67\cdot T_{\mathrm{amb}}}{T_{\mathrm{amb}}+243.5}}\cdot \mathrm{RH}\cdot 2.1674}{273.15+T_{\mathrm{amb}}}$.

### Statistical Analysis

The FTIR spectra shown in Figure 1b were normalized to the most intense peak at ≈1000 cm^−1^ for each spectrum, respectively. The full normalized spectra are shown in Figure S1, Supporting Information. The water contact angle presented in Figure 2b was determined by three separate measurements on the same substrate, and the presented value was mean ± SD. The spectra shown in Figure 3a,c were calculated by 1 − reflectance, without further statistical analysis. Figures 2a and 4c are raw data.

## Conflict of Interest

The authors declare no conflict of interest.

## Supporting information

Supporting Information

## Data Availability

The data that support the findings of this study are available from the corresponding author upon reasonable request.
