# Supplementary material for: From Chitosan to Chitin: Bio‐Inspired Thin Films for Passive Daytime Radiative Cooling
Source: Adv Sci (Weinh). 2023 Feb 15;10(11):2206616. doi: 10.1002/advs.202206616 (PMC10104647; doi:10.1002/advs.202206616)
Supplement: Supplementary file 1 — Supporting Information [file ADVS-10-2206616-s001.pdf]

Supporting Information

From Chitosan to Chitin: Bio-inspired Thin Films for Passive Daytime Radiative Cooling

*Tobias Lauster, Anika Mauel, Kai Herrmann, Viktoria Veitengruber, Qimeng Song, Jürgen Senker, Markus Retsch**

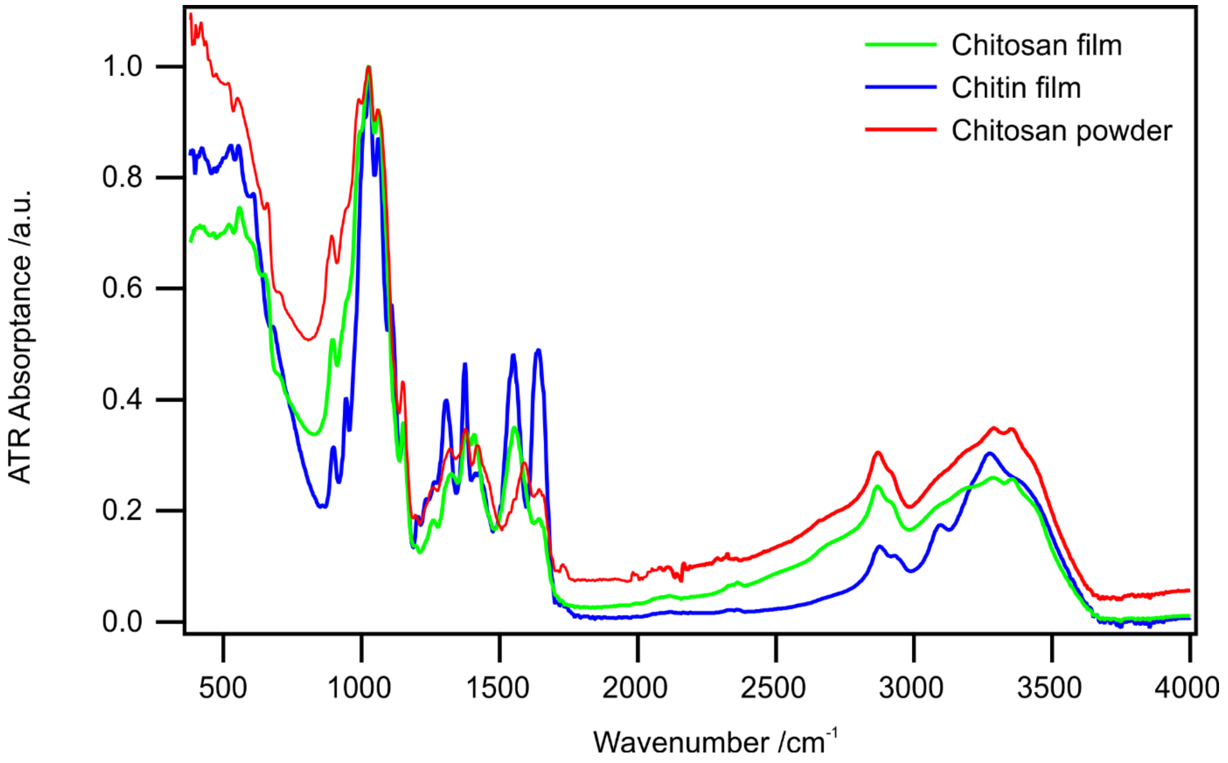

Figure S1. ATR-Absorptance of chitosan powder, a chitosan, and a chitin film. The spectra were normalized to the peak maximum at around 1000 cm^{-1} for better comparability.

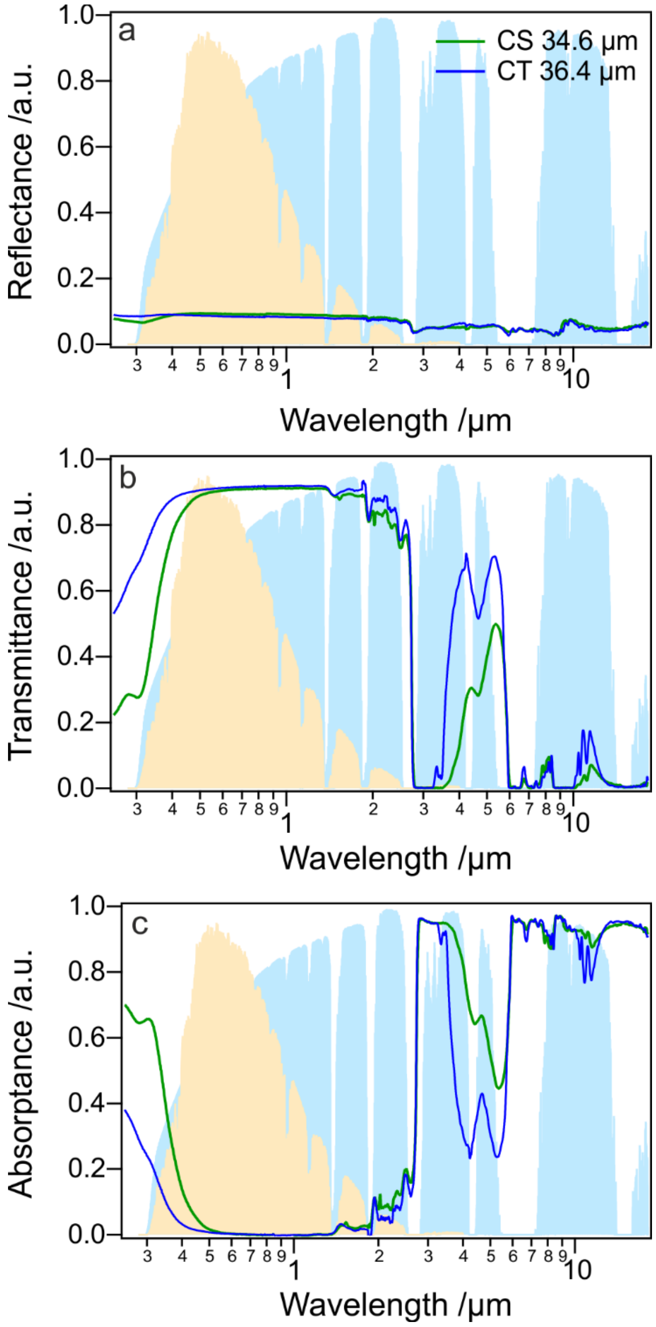

Figure S2. Reflectance (a), transmittance (b), and absorptance (c) spectra of a CS and CT film with comparable thickness. The spectra were collected from freestanding films without a silver substrate.

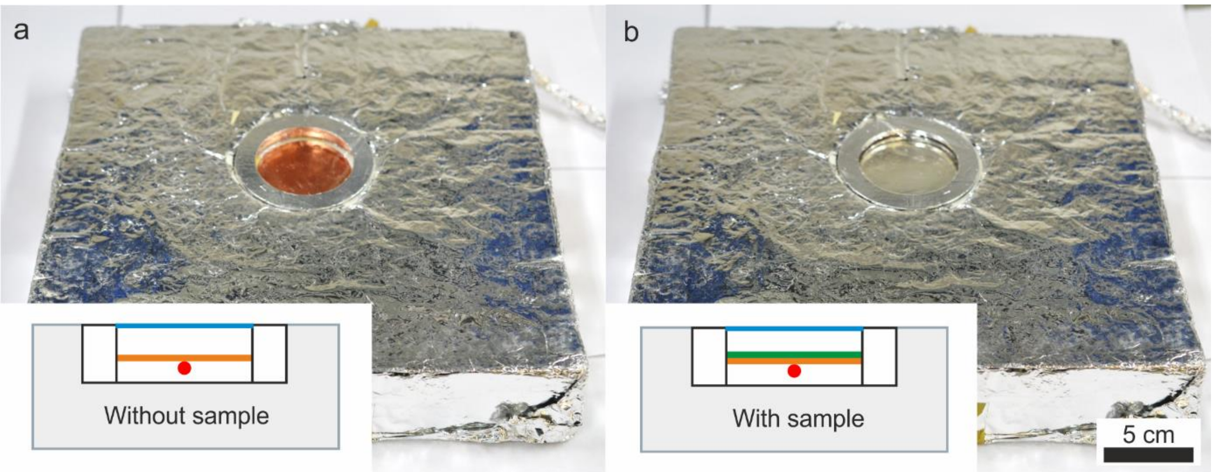

Figure S3. Photograph of the rooftop measurement setup (a) without sample and (b) with sample. The inset shows a schematic of the presented setup (as described in Figure 4 main text), respectively. The Styrofoam box is covered with Al foil to reflect sunlight and enclosed by PE foil to prevent convective losses. A copper plate visible in (a) is used to ensure good temperature distribution. The scale bar is 5 cm.

Table S1. Relative intensities I of the deconvoluted signals in the ^{13}C CP NMR spectra of chitosan powder, chitosan film, and chitin film samples. The sum of relative intensities of backbone carbons was normalized to six. Refinements were carried out with pseudo-Voigt lineshapes (Eq. 1) where δ_{iso} is the isotropic chemical shift, LB the line broadening, x the Gauss/Lorentz ratio, and I the relative intensity.

$$f(\delta) = I \cdot \left[(1-x)\frac{2\,\mathrm{LB}}{4\pi\cdot(\delta-\delta_{iso})^2+\pi\cdot\mathrm{LB}^2} + x \cdot \frac{\sqrt{4\ln(2)}}{\sqrt{\pi}\,\mathrm{LB}} \cdot e^{-\frac{4\ln(2)}{\mathrm{LB}^2}(\delta-\delta_{iso})^2}\right] \quad \text{(Eq. 1)}$$

Assignment	Acetyl group chitin		Chitosan and chitin backbone				Acetic acid	
	C=O	CH_3	C1	C2	C3 + C4 + C5	C6	C=O	CH_3
Shift	174 ppm	23 ppm	105 ppm	57 ppm	71; 83 and 75 ppm	61 ppm	180 ppm	25 ppm
Chitosan powder	0.046	0.119	1.056	0.939	3.014	0.991	/	/
Chitosan film	0.063	0.105	0.985	0.97	3.192	0.853	0.083	0.273
Chitin film	0.508	0.912	0.982	0.718	3.078	1.221	/	/
